# Safety Evaluation of Multiple Strains of *Lactobacillus plantarum* and *Pediococcus pentosaceus* in Wistar Rats Based on the Ames Test and a 28-Day Feeding Study

**DOI:** 10.1155/2014/928652

**Published:** 2014-10-14

**Authors:** Cheng-Chih Tsai, Sew-Fen Leu, Quan-Rong Huang, Lan-Chun Chou, Chun-Chih Huang

**Affiliations:** Department of Food Science and Technology, Hungkuang University, No. 1018, Section 6, Taiwan Boulevard, Shalu District, Taichung City 43302, Taiwan

## Abstract

Three lactic acid bacterial strains, *Lactobacillus plantarum*, HK006, and HK109, and *Pediococcus pentosaceus* PP31 exhibit probiotic potential as antiallergy agents, both in vitro and in vivo. However, the safety of these new strains requires evaluation when isolated from infant faeces or pickled cabbage. Multiple strains (HK006, HK109, and PP31) were subject to a bacterial reverse mutation assay and a short-term oral toxicity study. The powder product exhibited mutagenic potential in *Salmonella* Typhimurium strains TA98 and TA1535 (with or without metabolic activation). In the short-term oral toxicity study, rats received a normal dosage of 390 mg/kg/d (approximately 9 × 10^9^ CFU/kg/d) or a high dosage of 1950 mg/kg/d (approximately 4.5 × 10^10^ CFU/kg/d) for 28 d. No adverse effects were observed regarding the general condition, behaviour, growth, feed and water consumption, haematology, clinical chemistry indices, organ weights, or histopathologic analysis of the rats. These studies have demonstrated that the consumption of multiple bacterial strains is not associated with any signs of mutagenicity of *S.* Typhimurium or toxicity in Wistar rats, even after consuming large quantities of bacteria.

## 1. Introduction

Probiotics such as* Lactobacillus*,* Pediococcus*, and* Bifidobacterium* have been defined as “a live microbial feed supplement which beneficially affects the host animal by improving its intestinal microbial balance” [1]. Many strains of lactic acid bacteria (LAB) are typically regarded as safe because of their long history of use, and their status is generally recognised as safe [2]. In addition to demonstrating the efficacy of probiotics in improving human health, safety characteristics must be considered. For new isolate-specific species or strains of probiotics, novel probiotics cannot be assumed to share the historical safety of traditional strains [2, 3]. New or specific strains of probiotics are continually being identified. The efficacy of new strains should be carefully assessed prior to incorporating them into products, and a case-by-case evaluation should be conducted to determine whether they share the safety status of traditional food-grade organisms [3].

Various aspects associated with the safety of probiotic bacteria can be studied using in vitro and in vivo methods (e.g., the Ames test, animal models, and humans) [2, 3]. Numerous countries, such as European Community members, are developing more detailed guidelines regarding the regulations for novel and functional foods and related probiotic preparations. Conventional safety evaluation approaches, including those used for toxicological testing proposed by the Organization for Economic Cooperation and Development, are appropriate as an initial step in the evaluation of new probiotics [4]. Therefore, conducting a 28-day conventional rat-feeding study could be a useful starting point. The identification of substances that are capable of inducing mutations has also become crucial in safety assessments. The Ames test employs a biological assay for assessing the mutagenic potential of chemical compounds [5]. In addition, antimutagenicity is included among the functional properties of probiotic LAB [6].

*L. plantarum* HK006 was isolated from infant faeces, and* L. plantarum* HK109 and* P. pentosaceus* PP31 were isolated from pickled cabbage. These strains are catalase negative and Gram positive, and they exhibit the potential to suppress immunoglobulin E production through the induction of interleukin 12 (IL-12) and could thereby attenuate food allergies.* L. plantarum* has been used to reduce the allergenicity of soy flour [7].* P. pentosaceus* is a crucial industrial starter culture for fermenting foods, such as various meats, vegetables, and cheeses [8]. This study was conducted to evaluate the safety of multiple strains based on the methods recommended for the safety evaluation of novel probiotics. We confirmed the safety of the multiple-strain mixed powder product by using a bacterial reverse mutation assay and a 28-day feeding study on Wistar rats. We investigated the effects of the consumption of viable mixtures of multiple LAB strains on the health, growth, haematology, and blood chemistry in rats daily.

## 2. Material and Methods

### 2.1. Bacterial Strains, Culture Medium, and the Preparation of Multiple Lactic Acid Bacteria Strain Mixtures

Stock culture collections were maintained at −70°C in* Lactobacilli* MRS broth (DIFCO, Detroit, MI, USA) containing 25% glycerol. The cells were propagated twice in Lactobacilli MRS broth containing 0.05% L-cysteine by incubating the cells at 37°C for 20 h. The LAB powder (1 × 10^10^-1 × 10^11^ CFU/g) was produced by fermentation, freeze-dried (New Bellus Enterprise Co., Ltd., Tainan, Taiwan), and refrigerated at −20°C until it was needed for testing. The bacterial counts were determined by plating serial dilutions of the culture in phosphate buffered saline (PBS) or MRS agar. The plates were incubated anaerobically at 37°C for 48 h.

### 2.2. Ames Assay

*Salmonella* Typhimurium strains TA98 and TA1535 were purchased from the Bioresources Collection and Research Center (Hsin-Chu, Taiwan). Mutagenicity tests were conducted using* S*. Typhimurium strains TA98 and TA1535 and the S9 fraction as the metabolic activation system, as described previously [9]. The suspension mixture (total volume, 500 *μ*L) comprised of 4 mM NADP, S9 fraction (total protein, 170 *μ*g), 8 mM MgCl_2_, 33 mM KCl, 5 mM glucose-6-phosphate and phosphate buffer (pH 7.4), promutagen (NC, positive control, 0.4–4.0 *μ*g of BP, 2-AA, SA, or NQNO), test compound (5 mg of probiotic-combination mix), and 100 *μ*L of* S*. Typhimurium in overnight culture. The components were sequentially added to 2 mL of warm soft agar. The mixture was poured into a petri dish containing Vogel-Bonner minimal medium (1.5% agar in Vogel-Bonner E medium with 20 mg/mL glucose). After incubating for 2-3 d at 37°C, the revertant colonies (His^+^) were counted. The toxicity of the tested agents was assessed by observing the background bacterial growth on minimal agar plates caused by traces of histidine in the medium [10]. All of the experiments were performed in triplicate.

### 2.3. Animals and Experimental Design

These animal studies were approved by the Institutional Animal Care and Use Committee of HungKuang University, Taichung City, Taiwan (Approval number 96025). Forty-eight Wistar rats, aged 4-wk, were obtained from the National Laboratory Animal Centre (Taipei, Taiwan) and acclimatized to the laboratory conditions for 1 wk. At Day zero, the rats were randomly allocated to 3 groups (8 male, 8 female per group). A maximum of 2 rats were confined in each grid. The rats were identified by picric acid dye that was applied on the head or leg. The rats were provided diet and water ad libitum. A sterile gastric feeding tube was used for orally inoculating 2 of the 3 groups with mixed LAB strains in PBS at 2 doses; the control group received PBS only. For subsequent feeding, each rat received 1 mL of suitably diluted LAB suspensions for obtaining 9 × 10^9^ and 4.5 × 10^10^ CFU per kg body weight. The animal room was ventilated and maintained at 22°C ± 2°C with a relative humidity of 65% ± 5%. Artificial lighting was sequenced to provide 12 h light/dark cycles. The treatments lasted for 28 d; during this period, the activity, behaviour, and hair luster of each rat were observed and recorded daily. Water intake (WI) and feed intake (FI) were measured. The specific growth rate (SGR) was expressed as the average weekly weight gain (g). On Day 29, all the animals were sacrificed humanely, and blood and tissue samples were collected for further laboratory analysis.

### 2.4. Haematology, Blood Biochemistry, and Pathology

Blood samples were placed into ethylenediaminetetraacetic acid-treated tubes. Leukocyte, erythrocyte and platelet counts, haemoglobin concentration (HB), mean corpuscular volume (MCV), mean corpuscular haemoglobin (MCH), mean corpuscular haemoglobin concentration (MCHC), red cell distribution width, mean platelet volume, and haematocrit were determined by Dahua Medical Laboratory Centre (Changhua City, Taiwan).

After completing the haematology assays, the plasma was separated from the blood samples by performing centrifugation. Blood urea nitrogen (BUN), glutamic oxaloacetic transaminase (GOT), glutamic pyruvate transaminase (GPT), gamma-glutamyl transferase, total protein, albumin, urate, creatinine, total bilirubin, total cholesterol, alkaline phosphatase, Ca^2+^, Na^+^, K^+^, Cl^−^, and inorganic phosphate in the plasma were determined at Dahua Medical Laboratory Centre (Changhua City, Taiwan). A thorough necropsy was performed on all of the animals following euthanization. After dissection, the adrenals, testicles, heart, kidneys, liver, ovaries, and spleen were weighed (paired organs were weighed together). Organ-to-body weight ratios (relative organ weights) were calculated from the absolute organ weight and terminal body weight of the rats. Samples of the weighed organs were preserved in a neutral solution of 4% formaldehyde in PBS. Histopathologic analyses were conducted on 5 *μ*m sections of paraffin-embedded tissues from all of the control and high-dose animals after staining with haematoxylin and eosin. Additionally, histopathologic analyses were conducted on the adrenals, testicles, heart, kidneys, liver, ovaries, and spleen of the rats in all of the experimental groups.

### 2.5. Statistics

SGR and organ weights were evaluated using one-way analysis of variance (ANOVA) followed by Duncan's tests. The haematology and clinical chemistry were evaluated using ANOVA followed by Dunnett's tests. Statistical significance was indicated as a, b, and c for *P* values greater than 0.05.

## 3. Results

### 3.1. Ames Assay

**Figure 1:**
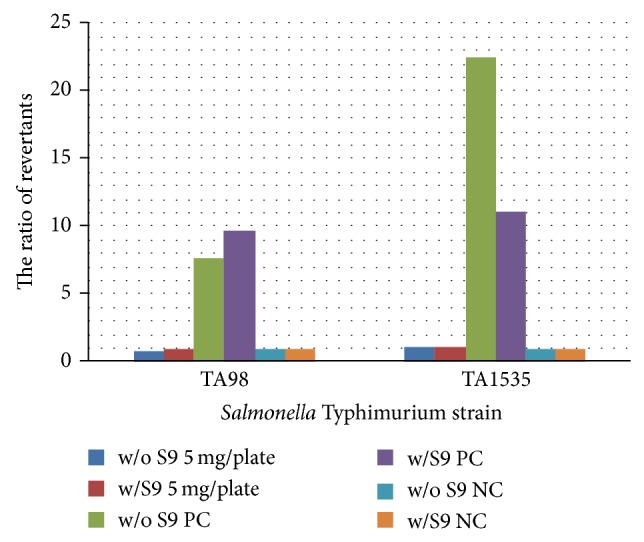
The influence of a probiotic-combination mix or promutagen on* Salmonella* Typhimurium. The reactions were separated with or without the addition of S9. Promutagens were added as a positive control group (with S9 addition, BP for TA98 and 2-AA for TA1535; without S9 addition, NQNO for TA98 and SA for TA1535). Sterile water was added as a negative control (NC). The ratio was defined as revertants in the reagent group: revertants in the NC group.

In this study, treatment with the probiotic combination mix did not cause mutagenicity of the* S*. Typhimurium strains, TA98 and TA1535, with or without metabolic activation (Figure 1). Promutagens caused mutagenicity of the* S*. Typhimurium strains TA98 (over 5-fold when compared with that of the negative control group) and TA1535 (over 10-fold when compared with that of the negative control group), with or without metabolic activation.

### 3.2. Feed, Water Intake, and Specific Growth Rate

No noticeable change was observed in the behaviour, activity, or hair luster of the rats in any of the groups throughout the experimental period. No diarrhea or other treatment-related sicknesses were recorded during the clinical observations. The daily FI or WI of the animals treated with various doses of mixed LAB strains did not differ significantly from those of the control group (data not shown).  Table 1 shows that the difference in the SGR of the treatment groups did not differ significantly from that of the control group (*P* > 0.05). Both the control and treatment groups exhibited a constant increase in SGR.

### 3.3. Haematological Parameters

Table 2 shows the effects of feeding various doses of mixed LAB strains on the haematological parameters. Haematological analysis revealed no treatment-related changes in the white or red blood cell counts, MCV, MCHC, or platelets among the rats in the various dosage groups and sex groups. However, significant increases were observed in the haemoglobin levels of the high-dose males in comparison with those of the other groups. Significant decreases were observed in haematocrit in the normal-dose males in comparison with those in the high-dose males, but no significant difference for the control group. The differences in haemoglobin or haematocrit were nonsignificant for the female groups. Significant increases were observed in MCH in the normal-dose females in comparison with that of the control group females. The difference in MCH among the male groups was nonsignificant. All values were within normal physiological ranges.

### 3.4. Clinical Chemistry

Clinical chemistry values at the terminal sampling time for the rats treated with normal or high-dose mixed LAB strains or those for the control groups indicated that no statistically significant differences existed in the plasma concentrations of BUN, GPT, total protein, albumin, Na^+^, Cl^−^, Ca^2+^, or phosphatase (Table 3). Significant decreases in alkaline phosphatase and K^+^ were observed in the normal- and high-dose females in comparison with those in the controls, but no dose-related responses were observed, and these differences occurred in one sex only. Significant increases in glucose concentrations were observed in the normal-dose and high-dose females in comparison with those in the control group. In the male rats, significant decreases in creatinine and cholesterol concentrations were observed in the normal- and high-dose groups in comparison with those in the control group, but they also exhibited a no dose-related response, and this occurred in one sex only. Significant increases in glucose concentrations were observed in the high-dose males in comparison with those in the normal-dose males, but there was no significant difference for the control group. Significant increases in K^+^ were observed in the high-dose males in comparison with those in the control group. The liver enzyme GOT decreased significantly in a dose-dependent manner for females.

### 3.5. Organ Weights and Pathology

**Figure 2:**
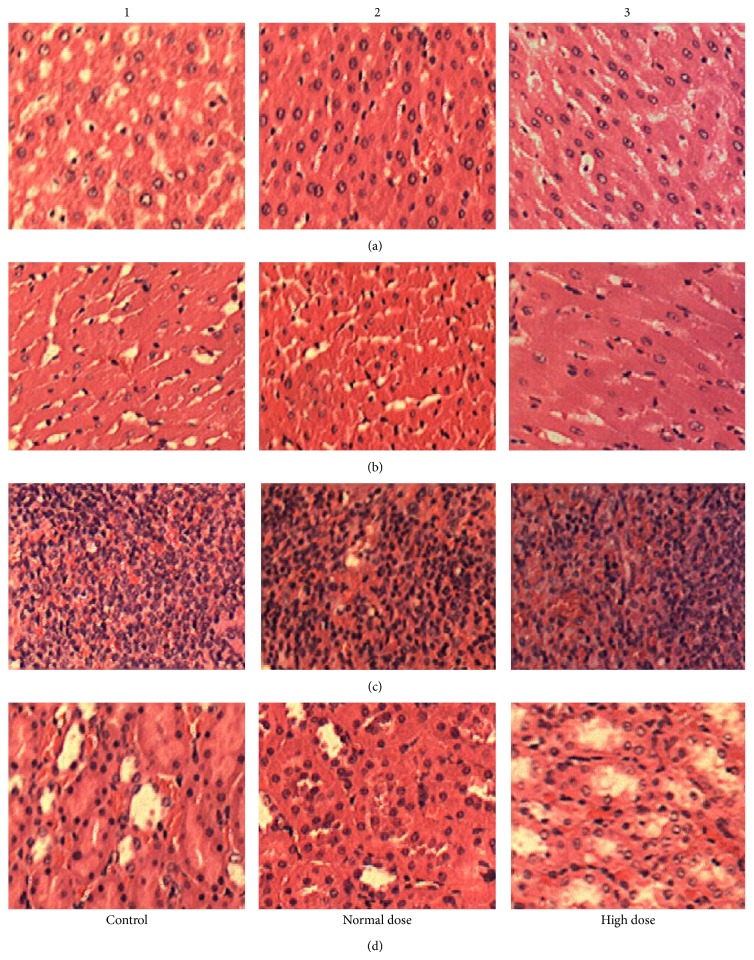
Histopathologic analysis included (a) liver, (b) heart, (c) spleen, and (d) kidney sections in male rats. Photomicrographs of transverse section of rats, column 1: control; column 2: normal dose; column 3: high dose multiple LAB strains (original magnification: ×100).

Table 4 shows the relative organ weights of the rats. There were no significant differences in the relative heart, kidney, testicle, or ovary weights among the rats in the various dosage groups or sex groups. The mean relative liver and spleen weights decreased significantly at normal and high doses in comparison with those of the male rats in the control group. The difference in the relative liver and spleen weights among the rats in the female groups was nonsignificant. Gross and microscopic examinations of the heart, liver, kidney, and spleen revealed no changes that could be attributed to the ingestion of various doses of the test strain (Figure 2). The tissues were morphologically normal and similar among the Wistar rats treated with the mixed LAB strains or PBS.

## 4. Discussion

Regarding the Ames test, the results from the promutagen treatment were similar to those reported by Zhang et al. [11] In this study, treatment with a probiotic-combination mix did not induce mutagenicity of the* S*. Typhimurium strains TA98 and TA1535, with or without metabolic activation, indicating that the probiotic-combination mix was free of mutagenic activity.

The 28-day administration of normal- and high-dose mixed LAB strains did not cause death or produce any clinical signs of toxicity. A significant increase was observed in the SGR of the rats treated using normal- and high-dose mixed LAB strains during the experimental period, whereas no significant difference was observed in the control group. Previous studies on LAB strains have demonstrated that certain* Lactobacillus* species, such as* L. acidophilus*,* L. pentosus*,* L. plantarum*,* L. reuteri*, and* Enterococcus faecium*, produce no oral toxicity in animals [12–17], but no researchers have evaluated the safety of multiple strains in a single product. For the safety of single strain,* L. plantarum* is widely in nature and has been found in fermented foods of both plant and animal origin [14]. Hirose et al. show that LP20 powder produced from heat-killed* Lactobacillus plantarum* L-137 is not acutely toxic (LD_50_ > 2,000 mg/kg bw) and no mutagenic/genotoxic effects were observed in the Ames, in vitro chromosome aberration, or in vivo micronucleus assays [14]. LP20 powder has potential ability to suppress immunoglobulin-E (IgE) production and the induction of interleukin-12 (IL-12) [14]. For the* P. pentosaceus* strain, there were few researches who had evaluated its safety with the Ames test and a 28-day feeding test.

Hepatomegaly and splenomegaly are usually indirect indicators of invasion and infection [18]. In this study, we did not observe macroscopic change in the liver or spleen morphology of the animals treated with the test strains in vivo. These results indicate that the rats experienced no infections resulting from the 28-day treatment with multiple LAB strains. Clinical chemical assays can be used to detect moderate to mild deficiency of nutrients or imbalances in nutrient metabolism, and these deficiencies are usually apparent before any clinical symptoms or changes in host body weight [18]. In this study, the relative weights and morphology of the major organs did not exhibit any macroscopic changes or significant differences. Furthermore, the animals in these groups exhibited similar daily FI and WI in comparison with those of the control group. No significant difference in SGR was observed between the treatment groups and the control group (*P* > 0.05).

Although statistical significance was observed for some parameters in this study (e.g., haematology, clinical chemistry, and organ weights), we hypothesised that none of these changes were attributable to the treatment because the changes remained within the range of historical controls, were present in only one sex, or were observed only sporadically. In similar studies [15, 19, 20], fluctuating results were observed in different feeding groups. However, these results did not affect the conclusions derived from the safety evaluation.

In summary, multiple strains of* S*. Typhimurium exhibited no evidence of mutagenic potential, with or without metabolic activation. A 28-day toxicity study was conducted, in which a repeated normal dosage of 390 mg/kg/d (approximately 9 × 10^9^ CFU/kg/d) and a high dosage of 1950 mg/kg/day (approximately 4.5 × 10^10^ CFU/kg/d) of bacteria administered to Wistar rats induced no abnormalities in external appearance, behaviour, FI, WI, or SGR. No significant toxicological effect was observed in the results of haematological and clinical chemical examinations, relative organ weights, or the macroscopic and histological data. In this case, the nonobserved adverse effect level of the mixed LAB strains was considered to be more than 1950 mg/kg/d. Therefore, multiple LAB strains (*L. plantarum* HK006 and HK109, and* P. pentosaceus* PP31) are nonpathogenic and safe for animal or human consumption.

## Figures and Tables

**Table 1:** The specific growth rate∗ (SGR) (mean ± S.D) of rats that were fed with multiple LAB strains at different doses for 28 days.

Dose	Week 1		Week 2		Week 3		Week 4	
	Male	Female	Male	Female	Male	Female	Male	Female
High	24.67 ± 9.26^a^	24.67 ± 9.26^a^	67.98 ± 7.06^a^	58.85 ± 7.03^a^	111.39 ± 11.98^a^	75.08 ± 6.75^a^	174.91 ± 23.06^a^	110.29 ± 8.4^a^
Normal	28.77 ± 1.53^a^	27.61 ± 1.72^a^	68.11 ± 5.26^a^	58.08 ± 5.26^a^	111.51 ± 8.1^a^	77.54 ± 6.53^a^	176.12 ± 13.44^a^	114.71 ± 10.06^a^
Control	30.36 ± 1.91^a^	28.16 ± 4.36^a^	69.96 ± 6.41^a^	60.01 ± 5.89^a^	110.64 ± 9.52^a^	81.35 ± 5.42^a^	175.57 ± 14.42^a^	116.61 ± 10.77^a^

^*^Specific growth rate = (*W* − *W*_0_)/*W*_0_ × 100 (%), *W*: rat weight on the defined feeding days; *W*_0_: rat weight on day 0.

*P* < 0.05 (Duncan's test). Statistical analysis was performed according to the procedures described in the Methods section. ^a^A significant difference of the mean between male or female rats fed with control or a medium or high dose of LAB was not observed.

**Table 2:** Haematological findings in rats that were treated orally with multiple LAB strains for 28 days.

Parameters		Control	Multiplex LAB strains	
			Normal dose	High dose
WBC (10^3^/*μ*L)	M	8.13 ± 0.84^a^	8.21 ± 1.12^a^	8.33 ± 0.92^a^
	F	7.29 ± 2.66^a^	9.5 ± 1.04^a^	7.93 ± 1.66^a^

RBC (10^6^/*μ*L)	M	6.84 ± 0.15^a^	6.64 ± 0.17^a^	6.8 ± 0.22^a^
	F	6.83 ± 0.41^a^	6.6 ± 0.31^a^	6.75 ± 0.2^a^

Haemoglobin (g/dL)	M	14.69 ± 0.25^a^	14.69 ± 0.18^a^	14.94 ± 0.19^b^
	F	14.15 ± 1.06^a^	14.4 ± 0.2^a^	14.54 ± 0.29^a^

Haematocrit (%)	M	44.38 ± 0.1^ab^	42.66 ± 1.24^a^	44.28 ± 1.6^b^
	F	42.64 ± 1.06^a^	41.8 ± 2.24^a^	42.71 ± 1.95^a^

MCV (fL)	M	64.33 ± 2.41^a^	64.25 ± 0.97^a^	65.18 ± 3.56^a^
	F	62.4 ± 2.16^a^	63.8 ± 5.77^a^	63.29 ± 2.72^a^

MCH (pg)	M	21.44 ± 0.71^a^	22.13 ± 0.18^a^	21.99 ± 0.72^a^
	F	20.7 ± 1.05^a^	21.9 ± 0.8^b^	21.55 ± 0.54^ab^

MCHC (%)	M	33.23 ± 0.57^a^	34.43 ± 0.9^a^	33.79 ± 1.4^a^
	F	33.21 ± 1.88^a^	34.5 ± 2.29^a^	34.08 ± 1.08^a^

Platelet (10^3^/*μ*L)	M	720 ± 53.84^a^	769.5 ± 146.9^a^	832.25 ± 154.2^a^
	F	707.75 ± 281.54^a^	774.3 ± 187.43^a^	780.38 ± 91.37^a^


All values are represented as the mean ± S.D. of Wistar rat (*n* = 7-8/sex/dose).

WBC: mean white blood cell; RBC: mean red blood cell; MCV: mean corpuscular volume; MCH: mean corpuscular hemoglobin; MCHC: mean corpuscular hemoglobin concentration; M: male; F: female.

^a,b^ *P* < 0.05 compared with the control group by ANOVA followed by Dunnett's test.

**Table 3:** Blood chemistry in rats that were treated orally with multiple LAB strains for 28 days.

Parameters		Control	Multiplex LAB strains	
			Normal dose	High dose
BUN (mg/dL)	M	8.24 ± 1.04^a^	8.91 ± 0.75^a^	8.15 ± 0.57^a^
	F	8.49 ± 1.36^a^	8 ± 0.98^a^	7.83 ± 0.78^a^

GOT (U/L)	M	106.25 ± 15.38^ab^	122.63 ± 20.63^a^	98 ± 17.73^b^
	F	139.38 ± 18.21^c^	120.3 ± 10.86^b^	93.25 ± 11.59^a^

GPT (U/L)	M	79.13 ± 6.13^a^	78.38 ± 15.01^a^	67.88 ± 11.31^a^
	F	51.13 ± 7.3^a^	51 ± 8.64^a^	53 ± 10.7^a^

Alkaline phosphatase (U/L)	M	92.38 ± 11.16^a^	91.63 ± 14.47^a^	87.5 ± 8.89^a^
	F	160.13 ± 45.01^b^	108.7 ± 8.64^a^	98.5 ± 14.39^a^

Total protein (g/dL)	M	7.14 ± 0.14^a^	7.11 ± 0.14^a^	7.1 ± 0.07^a^
	F	7.28 ± 0.25^a^	7.1 ± 0.24^a^	7.14 ± 0.14^a^

Albumin (g/dL)	M	4.1 ± 0.18^a^	4.01 ± 0.11^a^	3.95 ± 0.05^a^
	F	4.11 ± 0.22^a^	3.9 ± 0.24^a^	4.06 ± 0.11^a^

Total bilirubin (mg/dL)	M	0.74 ± 0.06^b^	0.64 ± 0.09^a^	0.7 ± 0.09^ab^
	F	0.76 ± 0.13^b^	0.7 ± 0.01^ab^	0.64 ± 0.09^a^

GGT (U/L)	M	22.25 ± 3.15^b^	17.38 ± 3.62^a^	17.13 ± 1.96^a^
	F	14.13 ± 1.36^a^	14.9 ± 2.12^ab^	16.5 ± 2^b^

Creatinine (mg/dL)	M	0.55 ± 0.07^b^	0.5 ± 0.02^a^	0.48 ± 0.04^a^
	F	0.5 ± 0.05^a^	0.5 ± 0.03^a^	0.49 ± 0.02^a^

Cholesterol (mg/dL)	M	107.25 ± 13^b^	93.63 ± 9.49^a^	88.25 ± 11.09^a^
	F	96.25 ± 8^a^	91.3 ± 78.16^a^	94.13 ± 9.98^a^

Glucose (mg/dL)	M	121.13 ± 24.01^ab^	108.75 ± 20.62^a^	134.75 ± 14.49^b^
	F	66 ± 19.34^b^	113.1 ± 25^a^	111.13 ± 22.63^a^

Na^+^ (mmol/L)	M	143.5 ± 3.25^a^	144.13 ± 2.03^a^	143.75 ± 2.44^a^
	F	145.88 ± 3.18^a^	143.9 ± 2.91^a^	144.25 ± 2.17^a^

K^+^ (mmol/L)	M	4.73 ± 0.21^b^	4.91 ± 0.18^ab^	4.95 ± 0.15^a^
	F	5.21 ± 0.18^a^	4.9 ± 0.2^b^	4.98 ± 0.14^b^

Cl^−^ (mmol/L)	M	108 ± 2.73^a^	109.13 ± 2.17^a^	108.88 ± 3.3^a^
	F	107.5 ± 3.25^a^	108.1 ± 3.29^a^	107.38 ± 2.34^a^

Ca^2+^ (mg/dL)	M	9.76 ± 0.169^a^	9.56 ± 0.26^a^	9.69 ± 0.19^a^
	F	9.38 ± 0.33^a^	9.6 ± 0.25^a^	9.59 ± 0.25^a^

P (mg/dL)	M	4.63 ± 0.4^a^	4.54 ± 0.28^a^	4.79 ± 0.19^a^
	F	4.34 ± 0.21^a^	4.3 ± 0.23^a^	4.48 ± 0.27^a^

All values are represented as the mean ± S.D. of Wistar rats (*n* = 8–10/sex/dose). BUN: blood urea nitrogen; GOT: serum glutamic oxaloacetic transaminase; GPT: serum glutamic pyruvate transaminase; GGT: gamma glutamyl transferase; Na^+^: sodium; K^+^: potassium. Cl^−^: chloride; Ca^2+^: calcium; P: inorganic phosphate; M: male; F: female. ^a,b,c^ *P* < 0.05 compared with the control group by ANOVA followed by Dunnett's test.

**Table 4:** Organ relative weights (%)^§^ in rats that were treated orally with multiple LAB Strains for 28 days.

Organ (%)		Control	Multiplex LAB strains	
			Normal dose	High dose
Heart	M	0.32 ± 0.04^a^	0.3 ± 0.03^a^	0.31 ± 0.03^a^
	F	0.31 ± 0.03^a^	0.32 ± 0.02^a^	0.34 ± 0.03^a^

Liver	M	3.63 ± 0.3^b^	3.28 ± 0.21^a^	3.36 ± 0.23^a^
	F	3.23 ± 0.22^a^	3.27 ± 0.36^a^	3.26 ± 0.21^a^

Kidney (L)	M	0.37 ± 0.04^a^	0.36 ± 0.02^a^	0.36 ± 0.02^a^
	F	0.37 ± 0.03^a^	0.35 ± 0.03^a^	0.32 ± 0.07^a^

Kidney (R)	M	0.36 ± 0.03^a^	0.34 ± 0.01^a^	0.35 ± 0.02^a^
	F	0.36 ± 0.02^a^	0.34 ± 0.02^a^	0.35 ± 0.01^a^

Spleen	M	0.26 ± 0.02^b^	0.22 ± 0.05^a^	0.22 ± 0.03^a^
	F	0.22 ± 0.02^a^	0.25 ± 0.03^a^	0.23 ± 0.02^a^

Testicle (L)	M	0.43 ± 0.04^a^	0.43 ± 0.05^a^	0.43 ± 0.05^a^

Testicle (R)	M	0.44 ± 0.04^a^	0.44 ± 0.04^a^	0.43 ± 0.05^a^

Ovary (L)	F	0.09 ± 0.02^a^	0.14 ± 0.05^a^	0.08 ± 0.03^a^

Ovary (R)	F	0.08 ± 0.02^a^	0.14 ± 0.06^a^	0.09 ± 0.02^a^

All values are represented as the mean ± S.D. of Wistar rats (*n* = 8–10/sex/dose).

M: male; F: female.

^a,b^ *P*≦0.05 compared with the control group by ANOVA followed by Duncan's test.

^§^Relative weight = (organ weight/body weight) × 100%.
